# ADP‐ribosylation: An emerging regulator of the epigenome

**DOI:** 10.1002/1878-0261.70317

**Published:** 2026-08-10

**Authors:** Cristel V. Camacho, Chenqian Liu, W. Lee Kraus

**Affiliations:** ^1^ Laboratory of Signaling and Gene Regulation, Cecil H. and Ida Green Center for Reproductive Biology Sciences University of Texas Southwestern Medical Center Dallas USA; ^2^ Division of Basic Research, Department of Obstetrics and Gynecology University of Texas Southwestern Medical Center Dallas USA

**Keywords:** ADP‐ribosylation (ADPRylation), chromatin remodeling complex, gene regulation, histone, histone modifying enzymes, transcription, transcription factor

## Abstract

ADP‐ribosylation (ADPRylation) is a post‐translational modification best known for its roles in DNA damage responses and cytoplasmic signaling, but it also serves important functions in epigenome regulation. In the nucleus, ADPRylation modulates chromatin structure and gene expression through the coordinated modification of histones and chromatin‐associated proteins. Although poly(ADP‐ribosyl)ation (PARylation) has dominated the field, particularly as therapeutic targets in DNA repair deficient malignancies, a gap remains in our understanding of how mono(ADP‐ribosyl)ation (MARylation)—mediated by mono(ADP‐ribosyl) transferases (MARTs)—functions as a discrete, site‐specific epigenomic mark. Nuclear MART‐mediated ADPRylation modulates the activity, localization, and complex assembly of epigenomic enzymes, and directly modifies histones to influence chromatin accessibility and transcriptional dynamics. These reversible modifications intersect with canonical epigenomic marks, enabling rapid, context‐dependent control of gene expression. Emerging studies further implicate dysregulated nuclear ADPRylation in cancer, where altered MARylation of chromatin regulators and transcription factors contributes to aberrant gene expression programs and may represent a novel class of therapeutic vulnerabilities. In this review, we synthesize emerging insights into nuclear ADPRylation, with a focus on MART‐mediated regulation of histones and chromatin enzymes, and discuss how this regulatory layer expands current models of epigenomic control in physiology and how its alterations drive oncogenesis, offering novel, nonsynthetically lethal avenues for targeted cancer therapy.

## Graphical abstract

ADP‐ribosylation has emerged as a dynamic epigenetic signaling mechanism that modifies histones and chromatin‐associated proteins. Through coordinated PARylation and MARylation, it integrates with other histone modifications to regulate chromatin structure, transcription factor activity, and gene expression, influencing genome function and disease. Schematic generated by ChatGPT and edited using Microsoft PowerPoint.

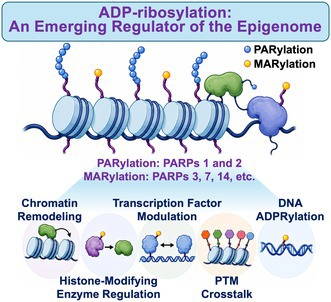

**Abbreviations**
ADPr‐ChAP: ADP‐ribose‐specific chromatin affinity purification
ADPRylation: ADP‐ribosylation
AhR: Aryl hydrocarbon Receptor
ARH3: ADP‐ribosyl‐acceptor hydrolase 3
asPARP1: Analog‐sensitive PARP1
CBP: CREB‐binding protein
ChIP: Chromatin immunoprecipitation
HDAC: Histone deacetylase
HPF1: Histone PARylation factor 1
IL‐4: Interleukin‐4
MARTs: Mono(ADP‐ribosyl) transferases
MAR‐Ub: ADP‐ribose‐ubiquitin modification
MARylation: Mono‐ADPRylation
PARG: Poly(ADP‐ribose) glycohydrolase
PARPs: Poly(ADP‐ribose) polymerases
PARylation: Poly‐ADPRylation
PRC2: Polycomb repressive complex 2
PTMs: Post‐translational modifications
β‐NAD^+^
: β‐nicotinamide adenine dinucleotide

## 1 Introduction

### 1.1 The post‐translational language of the cell

Biological signaling is not merely a product of protein abundance, but rather the result of rapid, reversible chemical modifications of proteins that expand the functional diversity of the proteome and are required for virtually every biological process. There are over 400 different types of post‐translational modifications (PTMs), ranging from the ubiquitous phosphorylation and ubiquitylation to the more context‐specific acetylation and methylation [1]. These PTMs serve as the language of the cell, dynamically affecting biochemical functions, protein stability, interactions, and localization [1, 2]. PTMs allow the cell to respond to environmental cues with temporal precision. Nowhere is this regulatory mode more critical than within the epigenomic landscape. In the nucleus, PTMs on histone tails and chromatin‐associated proteins dictate the structural state of chromatin, toggling between transcriptionally permissive euchromatin and silenced heterochromatin [3]. In malignant cells, this language is frequently altered, and this dysregulation of PTM patterns drives aberrant transcriptional programs that dictate cancer cell plasticity, therapeutic resistance, and immune evasion.

### 1.2 ADP‐ribosylation: An underappreciated epigenomic regulator

Among the diverse array of PTMs, ADP‐ribosylation (ADPRylation) stands out for its unique chemical complexity and energetic cost. This modification involves the enzymatic transfer of ADP‐ribose (ADPR) moieties from β‐nicotinamide adenine dinucleotide (β‐NAD^+^) to specific amino acid residues such as glutamate, aspartate, and serine [4, 5]. The modification exists in two principal forms: mono‐ADPRylation (MARylation), in which a single ADPR moiety is covalently attached to the substrate, and poly‐ADPRylation (PARylation), in which polymers of ADPR units are sequentially added through glycosidic ribose‐ribose bonds to generate linear or branched polymers on substrate protein [4, 5]. This duality is a major source of the functional diversity and regulatory complexity of ADPR signaling. These two modifications differ substantially in their biochemical and functional properties (Table 1). MARylation produces a discrete, site‐specific modification that can alter local protein conformation, catalytic activity, molecular interactions, or subcellular localization. In contrast, PARylation generates large, negatively charged polymers that function as multivalent scaffolds capable of recruiting numerous ADP‐ribose‐binding proteins, promoting biomolecular condensation, and rapidly remodeling protein complexes [4, 5, 6, 7]. Consequently, whereas PARylation often orchestrates broad, transient, high‐intensity signaling bursts such as those occurring during DNA damage repair (ADPR spray; see below), MARylation is generally thought to mediate more selective and context‐dependent regulation of individual protein functions (like a typical PTM).

**Table 1:** Biochemical and biophysical comparison of MAR and PAR.

Feature	MAR	PAR
Macromolecular structure	Single, discrete ADP‐ribose monomer	Long, linear or highly branched polymers (up to 200+ units)
Chemical linkages	Single glycosidic bond to amino acid chains (Ser, Asp, Glu, etc.)	Internal glycosidic α(1 → 2) ribose‐ribose bonds extending from the initial residue
Biophysical mechanism	Stereochemical alterations: modifies local surface chemistry to create or block protein–protein interaction surfaces	Electrostatic/steric disruption: concentrated negative charge drives local protein displacement and chromatin decondensation
Primary nuclear function	Persistent, context‐dependent rheostat for transcriptional programs and epigenomic enzyme kinetics	Acute, high‐intensity scaffolding for rapid recruitment of DNA repair complexes
Enzymatic writers	MARTs (e.g., PARP7, 10, 14, 16)	Polymerases (e.g., PARP1, 2)
Enzymatic erasers	Specialized mono‐hydrolases (e.g., MacroD1, MacroD2, TARG1, ARH1)	Glycohydrolases and endo‐hydrolases (e.g., PARG, ARH3)
Oncological paradigm	Epigenomic reprogramming, immune checkpoint suppression, and metabolic‐transcriptional adaptation	Maintenance of genomic stability, exploited via synthetic lethality (e.g., HR‐deficient cancers)

Abbreviations: ARH1/3, ADP‐(ribosyl)hydrolase 1 and 3; HR, homologous recombination; MARTs, [mono(ADP‐ribosyl) transferases]; PARG, Poly(ADP‐ribose) glycohydrolase; TARG1, ADP‐ribose glycohydrolase (OARD1).

This modification is catalyzed by the PARP family of enzymes, consisting of 17 distinct poly(ADP‐ribose) polymerases (PARPs) or mono(ADP‐ribosyl) transferases (MARTs), each with unique structural features, subcellular localizations, and substrate preferences [5, 6, 8]. The diversity of catalytic activities, regulatory inputs, and cellular contexts dramatically increases the complexity of PARP‐driven signaling pathways. Importantly, ADPRylation is increasingly recognized as a regulator of the epigenome. Beyond its well‐established roles in DNA damage responses, ADPRylation modulates chromatin structure and gene expression through the modification of histones, transcription factors, and chromatin‐associated enzymes. These activities enable ADPRylation to influence chromatin accessibility, transcriptional dynamics, and the integration of signaling pathways at the level of gene regulation. Crucially, while altered DNA repair has long been the focus of oncological ADPRylation research, the subtle, steady‐state alterations in gene transcription mediated by this PTM represent an emerging hallmark of tumor biology. In this review, we discuss the growing body of evidence supporting ADPRylation, particularly MARylation, as an epigenomic regulatory layer, with an emphasis on its roles in modulating chromatin‐associated proteins and shaping transcriptional programs.

### 1.3 Recentering ADPRylation in the nucleus

**Fig. 1:**
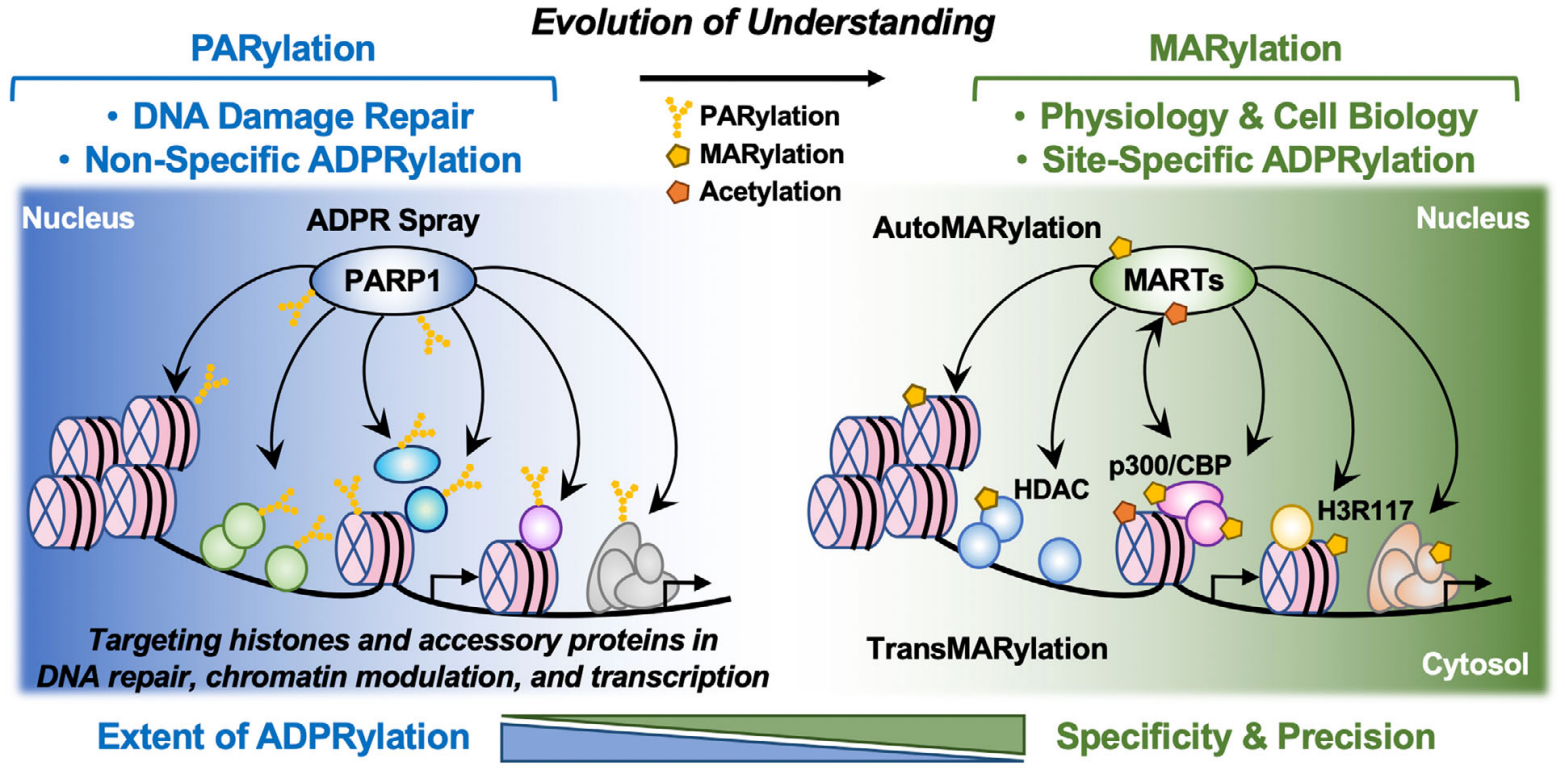
Evolution of understanding about ADPRylation: From PARylation signaling in DNA damage response to MARylation‐driven epigenomic regulation. This schematic illustrates the conceptual shift in the ADPRylation field from PARP1‐driven PARylation (events associated with rapid, extensive, and relatively non‐specific PAR chain formation [“ADPR spray”]) to MARylation mediated by various MARTs (more targeted and site‐specific signaling modality). Schematic generated using Microsoft PowerPoint.

As the prototypical and founding member of the PARP family, PARP1 and PARylation have historically dominated the field, primarily due to the substantial, rapid signaling bursts generated by PARP1 in response to DNA damage [9]. In this classic paradigm, the substantial production of anionic PAR chains, which we have described as ‘ADPR spray’, serves as a temporary scaffold to recruit repair factors and physically ‘shred’ or decondense chromatin at sites of injury and positions PARP1 as a central guardian of genome integrity (Fig. 1) [10, 11]. Over the past three decades, however, the field has expanded well beyond this DNA damage‐centric view. Recent advancements in chemical genetics and high‐resolution mass spectrometry have catalyzed a paradigm shift, recentering the field toward a view of ADPRylation as a constitutive and nuanced regulator of the epigenome [7, 12]. PARP1 is now widely appreciated for the fundamental roles that it plays in gene regulation, chromatin organization, and transcriptional control, functioning not only in acute stress responses but also in steady‐state cellular programs [9].

Even more compelling is the emerging evidence that MAR operates within the nucleus as a distinct regulatory mechanism with the same potential to modulate gene expression. We now understand that the majority of the 17‐member PARP family is actually MARTs that lack the catalytic machinery to extend polymers. Unlike the transient and bulky PAR chains, MARylation acts as a discrete, precision signaling modality rather than a bulky steric modulator. These single ADP‐ribose units are attached to specific amino acid side chains, altering the surface chemistry of nuclear proteins and modulating their interactome with high spatial precision (Fig. 1) [13, 14]. Hence, MAR is not merely a precursor to chain elongation, but a distinct and sophisticated signaling modality in its own right. Specifically, the presence of MARTs, such as PARP7 (TiPARP) and PARP10, within the nuclear compartment suggests a direct, yet less explored, link between MARylation and the modulation of the epigenome. Understanding how these enzymes target histones and transcriptional factors, and how they compete with other PTMs is the next frontier in decoding the ‘ADP‐ribose code’. A unified understanding of both PARylation and MARylation is essential to modern epigenomics, as these modifications represent a fundamental layer of control that bridges cellular metabolism with gene expression programs.

## 2 Nuclear MARTs as epigenomic regulators

The compartmentalization of MARTs within the nucleus is an actively regulated biological process, contradicting early assumptions that these enzymes were primarily cytoplasmic or associated only with the endoplasmic reticulum. Significant nuclear pools of PARP7 (TiPARP), PARP10, PARP12, and PARP14 have been identified, each possessing specialized domains for chromatin interaction [14, 15, 16]. For example, PARP7 is a zinc‐finger protein that directly binds DNA and is transcriptionally regulated by the Aryl hydrocarbon Receptor (AhR), positioning it as a key feedback regulator of xenobiotic and immune signaling [15, 16, 17]. PARP14, on the other hand, contains three macrodomains that allow it to ‘read’ pre‐existing ADPRylation marks, while its catalytic domain acts as a ‘writer’ of new MARylation events [18]. These enzymes do not simply float in the nucleoplasm; they are recruited to specific genomic loci, such as active enhancers and promoters, in response to physiological stimuli such as cytokine signaling or hormone exposure [19, 20]. The presence of these MARTs on chromatin provides a mechanism for the cell to integrate environmental cues directly into the transcriptional machinery, ensuring that epigenomic responses are both rapid and context dependent.

## 3 MARylation as a post‐translational regulator of chromatin enzymes

Nuclear MARTs exert their influence by modifying the very enzymes responsible for maintaining the epigenome, effectively acting as the ‘regulators of the regulators’. Through site‐specific MARylation, these enzymes can alter the activity, stability, and chromatin association of key epigenomic modifiers, including histone acetyltransferases, deacetylases, and chromatin remodelers. In this way, ADPRylation introduces an additional regulatory layer that fine‐tunes epigenomic enzyme function and shapes transcriptional outcomes.

### 3.1 Emerging links between PARP7 and p300

Emerging evidence suggests that PARP7 physically and functionally intersects with the histone acetyltransferase p300, a key regulator of chromatin‐dependent transcription and the epigenome [21]. These studies reveal a new layer of crosstalk between MARylation and lysine acetylation pathways in the nucleus. The functional intersection of PARP7 and the histone acetyltransferase p300 represents a sophisticated regulatory node within the nucleus, where MARylation and lysine acetylation converge. As a central transcriptional co‐activator, p300 facilitates gene activation by acetylating histone tails and nonhistone substrates, thereby lowering chromatin density and recruiting the transcriptional machinery. In contrast, PARP7 has emerged as a context‐dependent rheostat. Rather than acting as a simple ‘on/off’ switch, PARP7‐mediated MARylation appears to fine‐tune, restrain, or even reprogram p300‐driven transcriptional programs, positioning MARylation as a regulatory counterbalance within chromatin signaling networks. A recent study by Stokes *et al*. uncovered a metabolic–epigenomic link in which PARP7 functions as a sensor of nuclear NAD^+^ levels during adipogenesis [22]. Upon differentiation, nuclear NAD^+^ levels decline, leading to reduced PARP7 autoMARylation and consequent stabilization of the enzyme. This shift enables PARP7 to act as a transcriptional coregulator of C/EBPβ, where it promotes p300 activity and enhances histone acetylation across adipogenic gene loci [22]. As such, PARP7 acts as a molecular transducer, converting fluctuations in nuclear NAD^+^ levels into altered chromatin states through its reciprocal relationship with p300. Together, these findings position PARP7 as a metabolically responsive regulator that couples NAD^+^ availability to chromatin remodeling and transcriptional activation.

**Fig. 2:**
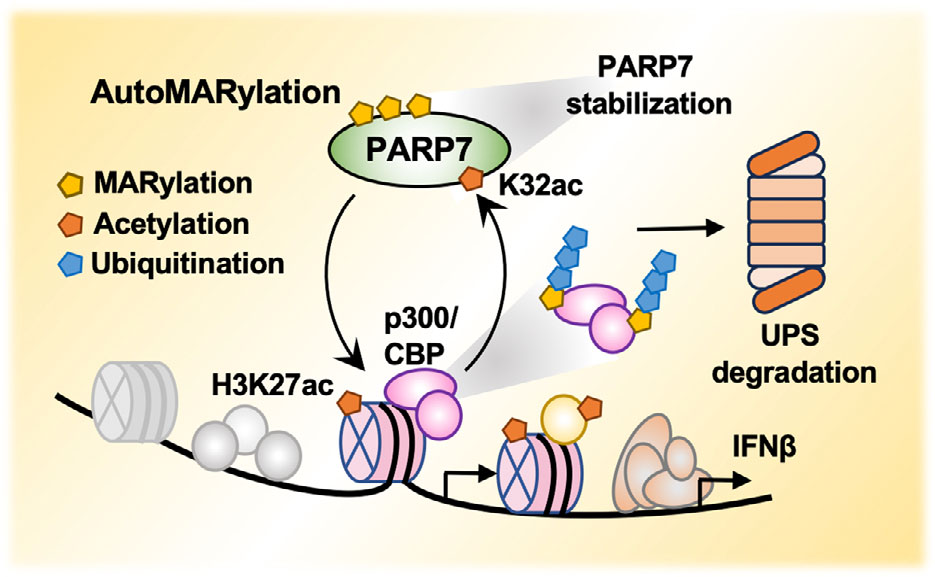
PARP7 functions in a complex bidirectional regulatory circuit with p300. PARP7 MARylates p300/CBP, promoting ubiquitylation and proteasome‐dependent (UPS) degradation of p300, thereby limiting the nuclear pool of acetyltransferase activity and reducing histone acetylation (e.g., H3K27ac). Conversely, p300 acetylates PARP7 at lysine 32 (K32ac), enhancing PARP7 protein stability and repressing IFN signaling [23, 24]. Schematic generated using Microsoft PowerPoint.

Further, two very recent paradigm‐shifting studies indicate that PARP7 and p300 directly modify each other [23, 24]. PARP7 has been shown to be a key suppressor of IFN‐signaling in tumor cells, a process that is dependent on PARP7 catalytic activity [25, 26, 27]. Two new studies have offered insights into the mechanisms by which PARP7‐mediated MARylation exerts its effects in this pathway. First, using an analog‐sensitive chemical genetics and proteomics approach, Siordia *et al*. identified key transcriptional regulators, including co‐activators and corepressors, as the most enriched substrates for PARP7. Among these, p300 and its immediate complex member CREB‐binding protein (CBP) were identified. Functional studies indicate that PARP7 MARylates p300/CBP, targeting p300 for ubiquitin‐mediated turnover, directly regulating the ‘pool’ of available acetyltransferase in the nucleus [23]. Second, Li *et al*. described the acetylation of PARP7 by p300 at lysine 32, which enhances the stability of PARP7, allowing it to exert its repressive effects in the TBK1‐IRF3‐STAT3 signaling axis [26] and attenuating IFN‐β‐induced transcription of interferon‐stimulated genes. Loss of PARP7 acetylation through p300 inhibition results in PARP7 proteasomal degradation and restoration of IFN signaling (Fig. 2) [24].

Thus, PARP7 functions in a complex bidirectional regulatory circuit. From a clinical standpoint, this bidirectional circuit between PARP7 and p300 represents a critical vulnerability in the tumor's ability to evade the host immune system. MARylation of p300 within its highly regulated CH3 or HAT domains could theoretically induce conformational changes that inhibit its acetyltransferase activity, representing a direct ‘PTM‐on‐PTM’ mode of regulation. In turn, p300‐mediated acetylation of PARP7 enhances stability and sustains its repressive function, establishing a reciprocal relationship in which each enzyme modulates the abundance and activity of the other. This dynamic interplay creates a finely tuned feedback loop that integrates ADPRylation and acetylation to control transcriptional outputs. More broadly, this circuit exemplifies how nuclear MARylation can function not only as a modifier of individual substrates but also as a regulator of epigenomic enzyme networks, ultimately shaping gene expression programs.

### 3.2 The PARP14 and histone deacetylase (HDAC) transcriptional switch

**Fig. 3:**
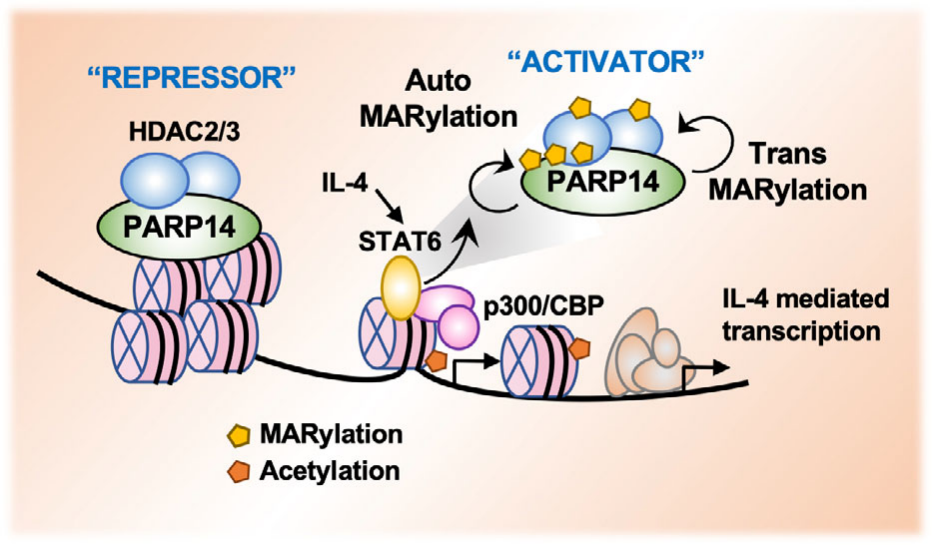
PARP14 functions as a MARylation‐dependent transcriptional switch. PARP14 regulates IL‐4‐dependent transcription by dynamically coordinating the balance between transcriptional repression and activation at STAT6 target genes. In the absence of IL‐4 stimulation, PARP14 is bound at STAT6‐responsive promoters, where it recruits histone deacetylases (HDAC2 and HDAC3) to maintain a repressive chromatin state. Upon IL‐4 signaling, PARP14 catalytic activity is stimulated, leading to autoMARylation and transMARylation of HDAC2/3. This modification disrupts HDAC association with chromatin, promoting their release and enabling the recruitment of co‐activators, increased histone acetylation, and transcriptional activation. Thus, PARP14 toggles between a repressive and activating role, functioning as a molecular switch that links MARylation activity to STAT6‐driven gene induction [20]. Schematic generated using Microsoft PowerPoint.

MARTs also interface with the repressive machinery of the cell. PARP14 has been shown to regulate interleukin‐4 (IL‐4)‐dependent transcription and has been characterized as a ‘transcriptional switch’ for STAT6‐mediated gene induction [20]. Mehrotra *et al*. demonstrated that PARP14 promotes the binding of STAT6 to its target genes, and this is dependent on both PARP14 expression levels and its catalytic activity [20]. PARP14 was found to directly associate with native STAT6‐responsive promoters in the absence of signaling. Without stimulation, PARP14 recruits histone deacetylases (HDACs) to repress transcription, but upon activation, its catalytic activity is triggered, resulting in PARP14 autoMARylation and HDAC 2 and 3 transMARylation, which effectively disrupts their association and promotes their release from promoters, allowing for the recruitment of co‐activators and gene induction [20]. Thus, PARP14 functions as a transcriptional switch, acting as a repressor in the absence of IL‐4 signaling through HDAC recruitment, then as an activator upon activation of MARylation activity through the release of HDACs (Fig. 3). In an oncology context, where chronic inflammatory signaling is a hallmark of many tumors, pharmacologic inhibition of PARP14 may represent a therapeutic strategy to modulate aberrant cytokine‐driven transcriptional programs and attenuate tumor‐promoting inflammatory signaling.

Further studies by Riley *et al*. demonstrated that PARP14 binds to specific DNA motifs in Th2 cells, regulating a broad set of cellular functions, including T‐cell‐associated functions such as Th1/Th2 polarization, T‐cell receptor signaling, and cytokine signaling [28]. Notably, PARP14 remains one of the few MARTs for which direct MARylation of HDACs has been demonstrated. Recent chemical‐genetic and proteomic studies have demonstrated that PARP14 directly MARylates a diverse repertoire of chromatin‐associated proteins, including histones, transcriptional coregulators, RNA‐processing factors, and chromatin remodeling proteins. These findings suggest that PARP14 regulates gene expression not only through protein–protein interactions with transcription factors such as STAT6, but also by directly modifying components of the chromatin regulatory machinery [29, 30]. While other PARP family members, particularly PARP1, have been shown to functionally antagonize chromatin repression by promoting the dissociation of transcriptional repressor complexes and inhibiting deacetylase activity (e.g., SIRT1), thereby facilitating chromatin relaxation and transcriptional activation [31, 32], direct regulation of HDACs by MARylation appears to be less well characterized. These observations suggest that PARP14 may represent a specialized example of a broader, yet underexplored, paradigm in which nuclear MARTs regulate the balance between acetylation and deacetylation to control transcriptional outcomes.

### 3.3 MART‐mediated modulation of the methylome

Compared to the well‐established roles of ADPRylation in regulating histone acetylation and deacetylation, its involvement in histone methylation pathways remains comparatively underexplored. Nevertheless, emerging evidence suggests that ADPRylation can influence the function of histone methyltransferases and demethylases through both direct and indirect mechanisms. PARP1, for example, has been shown to regulate Polycomb repressive complex 2 (PRC2) activity and H3K27 methylation [33, 34], as well as modulate the function of histone demethylases [35], highlighting the capacity of ADPRylation to shape methylation‐dependent chromatin states. Further, PARP1‐mediated ADPRylation of histone H2B at Glu 35 has been shown to influence methylation levels of histone H3, although the precise molecular mechanism underlying this crosstalk and its functional consequences for chromatin regulation and gene expression remain unclear [36]. In contrast, direct regulation of methylation enzymes by MARTs remains largely undefined. However, recent proteomic studies have identified methylation‐associated factors, such as PRMT1 [30, 37] and KMT2A [38], as candidate MART substrates, or SETDB1 as a direct interactor with PARP14 [39], suggesting that MARylation may represent an additional, previously underappreciated layer of control over histone methylation dynamics. Defining these mechanisms represents an important frontier in understanding how ADPRylation integrates into epigenomic regulatory networks. At present, however, these examples remain largely candidate‐based, and definitive MART‐dependent mechanisms controlling methyltransferase or demethylase activity remain limited. Establishing whether MARylation directly regulates methylome‐associated enzymes such as PRMT1, KMT2A, or SETDB1 will require site‐level mapping, biochemical validation, and functional chromatin assays.

### 3.4 Chromatin remodelers: MARTs at the level of nucleosome remodeling

ATP‐dependent chromatin remodeling complexes, including SWI/SNF (BAF), ISWI, CHD, and INO80 families, define the landscape of chromatin accessibility. While proteomics studies have demonstrated an enrichment of PARP1‐ADPRylated proteins within these complexes [40], a more nuanced regulatory layer is coming into focus. Emerging evidence suggests that site‐specific MARylation functions as a precision signaling modality that modulates the assembly and genomic targeting of these molecular machines. For example, Gibson *et al*. identified subunits of the BAF and CHD complexes as candidate targets identified by high‐resolution proteomics (e.g., the PARP3 ADPRylated proteome [41]). More recently, García Fernández *et al*. demonstrated that direct histone ADPRylation impacts chromatin architecture, triggering accessibility in the vicinity of DNA lesions [42]. In response to DNA damage, an early acute PAR signal is prevalent, followed by a more progressive and sustained MAR wave. The transient PAR serves to trigger chromatin loosening, while the maintenance of an open chromatin state is sustained by the more persistent MAR signal [42]. In these contexts, MARylation may act as a biochemical ‘scaffold’ to stabilize complex–DNA interactions or, conversely, as a ‘switch’ to prevent the premature recruitment of remodelers to specific enhancer elements. By tuning the activity and assembly of these master complexes, MARTs provide a layer of epigenomic plasticity that allows for rapid shifts in chromatin accessibility.

## 4 The MARylated nucleosome: Expanding the epigenomic landscape

Histone proteins are among the most abundant and diverse substrates for nuclear ADPRylation, with modifications identified on all four core histones (H2A, H2B, H3, and H4) and the linker histone H1 [40, 41, 43]. While PARylation of histones is a hallmark of DNA repair, where serine residues are the predominant acceptor sites, PARP1 and PARP2 can also generate histone MARylation at serine residues through their interaction with histone PARylation factor 1 (HPF1). HPF1 completes a composite active site with PARP1/2 and redirects acceptor specificity toward serine residues, making serine MARylation a major histone‐associated signal during the DNA damage response [44, 45, 46, 47]. Importantly, HPF1‐dependent serine MARylation should not be viewed solely as an initiation step toward PARylation chain formation. Current evidence supports a model in which HPF1 promotes serine‐linked MARylation, whereas extension into longer PARylation chains requires dynamic dissociation of HPF1 from the PARP1/2 complex [44, 48]. Thus, serine MARylation can function as a discrete signaling modification in its own right, expanding the repertoire of histone ADPRylation beyond classical PARylation‐dependent chromatin remodeling. Also, PARP3 has been reported to sense nicked nucleosomes and promote site‐specific MARylation of histone H2B at Glu 2, providing an example of MART‐dependent modification of chromatin in the context of DNA single‐strand‐breaks [49]. Beyond DNA repair, ADPRylation can also exert broader regulatory effects on chromatin. For example, Huang *et al*. demonstrated that PARP1‐mediated ADPRylation of histone H2B at Asp 51 plays an important regulatory role, suppressing p300‐mediated H2B acetylation genome‐wide [50]. These findings highlight how localized, site‐specific ADPRylation events can propagate to influence other histone modifications and reshape chromatin states at a systems level. Although this example is driven by PARP1‐dependent PARylation, it raises the intriguing possibility that site‐specific MARylation may similarly coordinate crosstalk with neighboring post‐translational modifications, thereby modulating chromatin function and gene expression in a highly targeted manner.

**Fig. 4:**
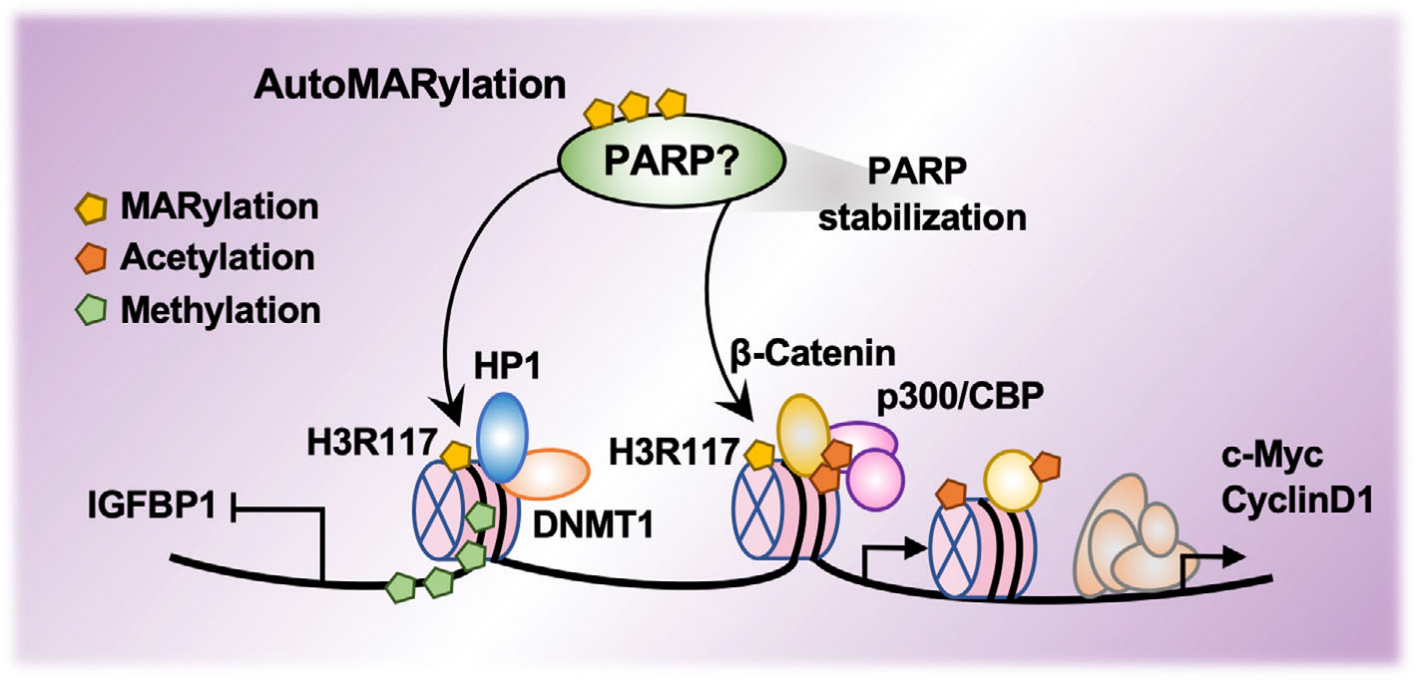
Histone H3R117 MARylation drives context‐dependent oncogenic transcriptional and epigenomic programs. This MARylation of histone H3 at Arg 117 (H3R117) regulates multiple cancer‐associated pathways in colorectal cancer cells. In one model, H3R117 MARylation is associated with p300, promoting its recruitment and/or activity toward β‐catenin, resulting in enhanced β‐catenin acetylation and increased expression of oncogenic targets such as c‐Myc and Cyclin D1, thereby supporting proliferation (*right*). In a distinct regulatory context, H3R117 MARylation promotes lipogenic reprogramming through repression of *IGFBP1*. This occurs via increased enrichment of repressive chromatin marks and factors, including H3K9me2, HP1, and DNMT1, at the *IGFBP1* promoter, leading to DNA hypermethylation and transcriptional silencing (*left*) [51, 52]. Schematic generated using Microsoft PowerPoint.

One such example is the histone H3 MARylation at Arg 117 (H3R117) [51]. Ling *et al*. first identified this MARylation through mass spectrometry using LoVo cells and found the modification to promote proliferation of this colon cancer adenocarcinoma cell line. H3R117 MARylation was found to be associated with p300. Although a clear mechanism was not proposed, it is suggested that H3R117 MARylation enhances p300 recruitment/interaction and activity toward β‐catenin, leading to its hyperacetylation, which in turn upregulates the expression of key oncogenic proteins c‐Myc and CyclinD1 [51]. More recent work by Wang *et al*. indicates that H3R117 also promotes lipogenesis in colorectal cancer, mediated through IGFBP1 [52]. In this case, the MARylation of H3R117 increased H3K9me2, HP1, and DNMT1 enrichment at the *IGFBP1* promoter, resulting in hypermethylation of the locus and decreased expression of IGFBP1 in LoVo cells (Fig. 4) [52]. Despite these advances, a critical gap remains: the specific PARP enzyme responsible for catalyzing this histone MARylation event has not been definitively identified. This limitation hinders mechanistic insight into how these modifications are regulated, how they are targeted to specific genomic loci, and how they integrate with other epigenomic pathways. Given the diversity of the PARP/MART family and their distinct nuclear functions, it is likely that multiple enzymes contribute to histone MARylation in a context‐dependent manner. Defining the enzymes responsible for these marks represents an important and largely unexplored frontier. Moreover, identifying the ‘writer’ for H3R117 MARylation is a critical prerequisite for understanding this modification. Addressing these gaps will be essential for establishing histone MARylation as a bona fide epigenomic mechanism and for understanding how this modification shapes chromatin states and gene expression programs in both normal physiology and disease.

## 5 Mapping the ADPRylated genome: Defining the epigenomic landscape of ADPRylation

The discovery of ADPRylation as a reversible modification not only on proteins but also on DNA itself has significantly expanded our understanding of the epigenomic landscape. PARP1, PARP2, and PARP3 can ADPRylate phosphorylated DNA strand‐break termini, including 5′‐ and 3′‐terminal phosphate groups, in a break‐structure‐dependent manner [53, 54, 55, 56, 57]. Although the biological functions of DNA ADPRylation are still being defined, current evidence suggests that it may influence DNA repair pathway choice, break processing, and the coordination of chromatin‐associated repair events. Thus, DNA ADPRylation expands the epigenomic scope of ADPR signaling from protein‐based chromatin regulation to direct chemical modification of nucleic acid structures at sites of genome damage. Moreover, defining where MARylation and PARylation occur throughout the genome is essential for understanding how ADPRylation regulates DNA repair, transcription, chromatin accessibility, and higher order genome organization. Importantly, a complete mechanistic understanding of how ADPRylation shapes the epigenome will require high‐resolution genomic mapping of ADPRylation marks together with their corresponding ‘writers’, ‘erasers’, and ‘readers’, as well as systematic interrogation of how these landscapes respond to chemical inhibition of PARP/MART activity. Such integrative approaches will enable the prediction of functional regulatory mechanisms and reveal how site‐specific ADPRylation contributes to gene control across diverse biological contexts.

### 5.1 Initial genomic mapping to study chromatin‐associated ADPRylation

To address this challenge, researchers have developed a growing array of affinity purification and enrichment‐based genomic technologies, primarily centered around chromatin immunoprecipitation (ChIP) and related methodologies. Early breakthroughs established foundational maps of chromatin‐associated ADPRylation. ‘Click‐ChIP‐seq’, which combines analog‐sensitive PARP1 (asPARP1) with clickable NAD^+^ analogs, demonstrated that PARP1‐dependent ADPRylation is enriched at transcriptionally active promoters and colocalizes with PARP1, NELF, and RNA Polymerase II [41]. In addition, ADP‐ribose‐specific chromatin affinity purification (ADPr‐ChAP), utilizing the Af1521 macrodomain and WWE domain, enabled genome‐wide analysis of ADPRylation in response to oxidative stress induced by H_2_O_2_ [58]. Recent mapping efforts using PAR ChIP‐seq have highlighted the protective role of ADPRylation at genomic ‘hotspots’. PAR ChIP‐seq identified neuronal enhancers as major sites of ADPRylation, marking them as critical zones for endogenous DNA single‐strand break repair [59]. This protective role is further coordinated by kinases such as ATM, modulating PARylation levels to prevent the accumulation of RNA–DNA hybrids, thereby ensuring the high‐speed transcriptional activity does not compromise genome integrity [60]. Collectively, these studies provided the first evidence that ADPRylation is spatially organized across the genome and closely linked to active transcriptional regulation.

As the field evolved, the functional role of ADPRylation as a structural and regulatory chromatin scaffold became increasingly apparent. PAR ChIP‐qPCR studies demonstrated that PARylation stabilizes CTCF occupancy and preserves higher order chromatin looping required for maintenance of viral latency [61]. Similarly, in androgen receptor signaling, ADPRylation promotes the assembly of transcriptional coregulatory complexes through coordinated interactions between ADPR ‘writers’ and ‘readers’ [62]. Emerging studies have also begun to uncover extensive crosstalk between ADPRylation and other epigenomic pathways. A notable example is the MARylation of histone H3R117, which facilitates the recruitment or ‘trapping’ of the DNA demethylase TET1, linking histone ADPRylation to DNA methylation dynamics [63]. In addition, other sites of histone ADPRylation (e.g., H2BE35 and H2BD51) have been shown to be potent regulators of nearby AMPK‐mediated histone phosphorylation [36] and p300‐mediated histone acetylation [50], respectively. For the latter, these effects were observed on a genome‐wide scale [50]. Beyond the nucleus, ADPr‐ChAP‐seq mapping of mitochondrial nucleoids identified a specialized mitochondrial ADPRylation landscape regulated by NEURL4 and mitochondrial PARP1, implicating ADPRylation in mitochondrial transcriptional control and metabolic homeostasis [64, 65].

### 5.2 Technical challenges in mapping the ADPRylated genome

Despite these pioneering advances, major technical and conceptual challenges continue to limit a comprehensive understanding of the genomic landscape of ADPRylation. Many existing studies rely on conditions of acute PARP hyperactivation (e.g., treatment with H_2_O_2_), leaving basal and physiological ADPRylation landscapes poorly defined. The common use of formaldehyde in ChIP‐based approaches can also be problematic because it can lead to spurious activation of PARP1 and large increases in PAR accumulation [66]. In addition, the inherently transient and labile nature of the ADP‐ribose bond complicates chromatin‐based assays, as standard lysis and sonication procedures can lead to rapid loss of the modification. Strategies such as pretreatment with PARG inhibitors prior to chromatin isolation, as described by Wu *et al*. [59], may improve preservation of ADPRylation during genomic workflows.

Importantly, most current enrichment approaches rely on broad ADP‐ribose binding reagents or pan‐ADP‐ribose antibodies that do not reliably distinguish between MARylation and PARylation, severely limiting our understanding of MART‐specific chromatin regulation. Although several pan‐MAR detection reagents have recently been developed, including engineered ADP‐ribose‐binding macrodomains and MAR‐selective antibodies (some that specifically recognize modified Ser residues) [67, 68, 69, 70, 71], these reagents have not yet been systematically validated for genome‐wide chromatin profiling applications, and their performance in assays such as ChIP‐seq remains largely unexplored. Furthermore, because ADPRylation is generally a low‐abundance modification, ChIP‐based enrichment strategies often suffer from high background signal, low library complexity, and elevated duplication rates. Emerging technologies that are independent of biochemical enrichment, such as cleavage under targets and tagmentation (CUT&Tag) [72] and related low‐background chromatin profiling methods, may help overcome these limitations and substantially improve sensitivity and resolution. They also have the advantage of eliminating formaldehyde crosslinking.

### 5.3 Future directions toward high‐resolution ADPRylation epigenomics

Thus, having progressed from bulk biochemical observations to increasingly sophisticated genomic landscapes, the next phase of discovery will require the development of high‐resolution mapping technologies capable of resolving site‐specific MARylation and PARylation together with their associated regulatory enzymes. Integrating genomic localization data with proteomics, chemical biology, and pharmacological perturbation studies will be essential for defining how ADPRylation governs transcriptional programs and chromatin states at the systems level. Ultimately, comprehensive genomic mapping of ADPRylation, its writers, erasers, and downstream effectors will be necessary to establish mechanistic frameworks linking ADP‐ribose signaling to epigenetic regulation and genome control.

## 6 PTM‐on‐PTM regulation: ADPRylation as a modulator of protein signaling

ADPRylation does not function in isolation but instead operates within a highly interconnected network of post‐translational modifications. Proteome‐wide analyses have revealed that ADPRylation sites frequently occur in close proximity to other regulatory PTMs, particularly phosphorylation [40, 41]. Notably, Gibson *et al*. demonstrated that phosphorylation sites, and to a lesser extent other PTMs, are often located at or near ADPRylation sites across the proteome, suggesting that ADPRylation is strategically positioned at hubs of regulatory activity [41]. These findings suggest a high degree of crosstalk and support a model in which ADPRylation functions both as a local modulator of protein activity and as a coordinator of signaling crosstalk, particularly in cooperation with kinase‐driven pathways.

A striking example of this interplay is provided by studies of histone H2B, where PARP1‐mediated ADPRylation at Glu 35 directly influences phosphorylation of the adjacent Ser 36 residue during adipogenesis [36]. Huang *et al*. showed that ADPRylation at H2B Glu 35 blocks phosphorylation at Ser 36, thereby repressing transcriptional programs required for adipocyte differentiation [36]. This work highlights a precise, site‐specific mechanism by which ADPRylation can regulate neighboring PTMs to control chromatin function and gene expression.

A recently described example of PTM crosstalk is the hybrid ADP‐ribose‐ubiquitin modification (MAR‐Ub), in which ubiquitin is conjugated directly to a protein‐linked ADP‐ribose moiety rather than to a canonical lysine residue on the protein substrate. Earlier studies linked ADPRylation to ubiquitin‐dependent signaling through the recognition of ADP‐ribose structures by WWE domains [73, 74]. Subsequent biochemical and structural studies showed that DELTEX family E3 ligases can engage ADP‐ribose as a substrate for ubiquitin transfer, binding to the MAR on the substrate proteins through their DTC domains and ubiquitylating MARylated substrates through their RING domains [75, 76, 77, 78]. The chemical nature of this hybrid modification was further defined by the demonstration that ubiquitin is directly linked to protein‐bound MARylation through an ester bond, establishing MAR‐Ub as a distinct hybrid PTM [79]. The development of nonhydrolyzable MAR‐Ub reagents enabled the identification of RNF114 as a MAR‐Ub reader, supporting the existence of dedicated recognition mechanisms and suggesting a broader network of MAR‐Ub writers, readers, and erasers [80]. Given that histones are established substrates for both ADPRylation and ubiquitylation and that both modifications regulate transcription, DNA repair, and chromatin structure [81], MAR‐Ub may add an additional layer of complexity to chromatin‐associated signaling. However, the genomic distribution, regulatory enzymes, and functional consequences of MAR‐Ub on chromatin remain important areas for future investigation.

While studies have largely focused on PARylation mediated by PARP1, they raise the intriguing possibility that similar crosstalk mechanisms may be mediated by MARylation. Given the site‐specificity and emerging regulatory roles of MARylation, it is plausible that MARTs likewise influence adjacent PTMs such as phosphorylation, acetylation, or methylation to fine‐tune protein function and chromatin dynamics. However, direct evidence for such mechanisms remains limited. Defining how MARylation interfaces with other PTMs represents an important and largely unexplored frontier, with the potential to reveal new principles of combinatorial signaling in gene regulation.

## 7 A tunable signal: Dynamic control of MARylation by hydrolases

A hallmark of a functional epigenomic signaling system is reversibility, and ADPRylation is a highly dynamic and reversible post‐translational modification, regulated not only by PARP ‘writers’ but also by a diverse set of hydrolases that function as ‘erasers’. The reversibility of this pathway is primarily controlled by ADP‐ribosyl‐acceptor hydrolase 3 (ARH3), which acts as a major hydrolase for serine‐linked MARylation. Whereas poly(ADP‐ribose) glycohydrolase (PARG) efficiently hydrolyzes ribose‐ribose linkages within PAR chains, ARH3 removes terminal serine‐linked MAR, thereby completing the HPF1‐PARP1/2‐ARH3 regulatory axis described above [82, 83, 84, 85]. Proteome‐wide studies further support this model by showing that HPF1 and ARH3 exert opposing effects on the cellular serine ADP‐ribosylome, with HPF1 promoting and ARH3 reversing this modification across many nuclear substrates [86]. Incorporating this axis is important for distinguishing serine MARylation from both classical PARylation and MART‐dependent MARylation, and for understanding how histone ADPRylation is dynamically installed and removed at chromatin. In contrast, ADPRylation on other acidic residues, such as glutamate‐ and aspartate‐linked MARylation, is reversed by a distinct set of macrodomain‐containing hydrolases. MacroD1, MacroD2, and TARG1/OARD1 have been shown to hydrolyze ester‐linked ADP‐ribose from glutamate and aspartate residues and are therefore the primary candidate erasers for acidic‐residue ADPRylation on histones and other chromatin‐associated proteins [87, 88]. Thus, different ADPR acceptor chemistries are controlled by distinct eraser systems: ARH3 primarily reverses serine‐linked MARylation, whereas MacroD1, MacroD2, and TARG1/OARD1 regulate glutamate/aspartate‐linked ADPRylation. This distinction is important for understanding how histone ADPRylation is dynamically installed and removed at chromatin.

These enzymes ensure tight control over ADP‐ribose signaling and are essential for maintaining proteome and chromatin homeostasis. The rapid turnover of MARylation marks mediated by these erasers allows the cell to maintain a high level of temporal control over its transcriptional responses. An intriguing example of integrated ADPRylation signaling is provided by PARP14, which has been shown to function as a writer, reader, and eraser of MARylation [18]. In this study, Torreta *et al*. demonstrated that PARP14 catalyzes MARylation of target proteins through its C‐terminal catalytic domain, while its tandem macrodomain modules enable it to recognize and bind ADP‐ribose modifications. Strikingly, one of these macrodomains also exhibits hydrolase activity, allowing PARP14 to remove MAR from substrates without amino acid selectivity and no activity toward PAR [18]. This multifunctional architecture enables PARP14 to both install and interpret MARylation marks, as well as dynamically reverse them, positioning PARP14 as a self‐contained regulator of ADP‐ribose signaling. Such coupling of writing, reading, and erasing activities within a single protein suggests a highly localized and tunable mode of regulation, in which PARP14 can precisely control the timing, amplitude, and specificity of MARylation‐dependent signaling events.

Because the writers of these marks (PARPs and MARTs) use NAD^+^ as their only substrate, the entire system is intrinsically linked to cellular metabolism. Fluctuations in nuclear NAD^+^ levels, driven by diet, aging, or stress, directly modulate the intensity of the ADPRylation signal. This integration allows the cell to sense its energetic health and translate that information into broad changes in the epigenome. For example, high NAD^+^ levels may promote a permissive ADPRylated chromatin state that favors rapid gene induction, while NAD^+^ depletion could lead to the loss of these marks and a shift toward epigenomic silencing.

Despite the identification of multiple MAR hydrolases, their substrate specificity, genomic targeting, and coordination with individual PARP enzymes remain poorly understood. In particular, it is largely unknown how hydrolases are recruited to specific chromatin loci or how they selectively reverse MARylation on defined nuclear substrates. This gap is especially relevant in the context of MART‐driven signaling, where site‐specific MARylation is proposed to regulate transcription factors and chromatin regulators. Defining how MAR hydrolases interface with nuclear PARPs will be essential for understanding the dynamic regulation of ADPRylation and for establishing MARylation as a fully reversible and tunable epigenomic signal.

## 8 The next frontier: Translating MARylation of the genome into clinical action

While the clinical success of PARP1/2 inhibitors has revolutionized the treatment of *BRCA1/2*‐mutant cancers through synthetic lethality, the therapeutic exploitation of MARTs represents a distinct and burgeoning frontier. Several high specificity and high potency chemical inhibitors of MARTs have been developed, including inhibitors of PARPs 7, 10, 11, 14, and 16 (Table 2), all of which are ≥ 10‐fold more potent for the intended MART versus other family members. The highly selective inhibitors of PARP7, PARP14, and PARP16 have been used to explore the biology of MARTs in various biological systems [25, 89, 90, 91, 92, 93, 94, 95, 96, 97]. The dysregulation of nuclear MARTs is a common feature in cancer, inflammation, and metabolic disorders, making them promising targets for therapeutic intervention. In oncology, PARP7 and PARP14 are often overexpressed and utilized by tumor cells to suppress the cGAS‐STING‐Interferon pathway, thereby evading antitumor immunity [20, 26]. The development of selective small‐molecule inhibitors for these enzymes, such as the PARP7 inhibitor RBN‐2397, has shown significant promise in preclinical models by restoring the production of type I interferons and enhancing the recruitment of T cells to the tumor [19, 23, 25].

**Table 2:** The next frontier of MART inhibitors.

Target	Inhibitor	Status	Mechanism	Ref
PARP7	RBN‐2397	Phase I	Stimulates Type I IFN response, targets AHR signaling	[25]
PARP14	RBN‐3143	Phase I	Suppression of inflammatory signaling	[89]
PARP14	RBN‐012579	Preclinical	Modulates macrophage polarization, protein homeostasis, stress granule dynamics	[90, 91, 92]
PARP10	OUL232	Preclinical	Modulates cell death	[93]
PARP10	OUL35	Preclinical	Sensitizes cells to genotoxic stress	[94]
PARP11	ITK7	Preclinical	Regulates nuclear envelope integrity	[95]
PARP16	DB008	Preclinical	Nutrient stress and protein homeostasis	[96, 97]

*Note:* Table summarizing current efforts to pharmacologically target members of the MART family, highlighting selective inhibitors, their stage of development, and proposed mechanisms of action. The therapeutic landscape for MARTs is still in its early stages, underscoring the need for further mechanistic and translational studies to fully exploit their potential in disease contexts.

Beyond cancer, the role of PARP14 in Th2‐type immune responses positions it as a potential target for treating allergic asthma and other chronic inflammatory conditions [20, 28]. To date, selective inhibitors targeting PARP7 and PARP14 have advanced to Phase I clinical trials [25, 90, 91], highlighting a growing interest in therapeutically drugging the MARTs. However, these efforts represent only an initial step, and substantially more work is needed to fully exploit the therapeutic potential of MARTs (Table 2). Importantly, targeting nuclear MARTs offers a distinct clinical advantage over traditional PARP1/2 inhibitors, which primarily target DNA repair and can cause significant hematological toxicity. Further, MART inhibitors are increasingly viewed as immunomodulatory and epigenomic agents. Hence, by selectively inhibiting MART‐mediated epigenomic regulation, it may be possible to reprogram the transcriptional landscape of diseased cells with higher precision and fewer side effects. For example, by disrupting the MARylation‐dependent brakes on transcriptional programs, such as the PARP7‐mediated suppression of interferon signaling or PARP14‐driven oncogenic scaffolding, these small molecules may reprogram the tumor microenvironment. These agents offer the potential to not only arrest tumor growth directly but also to sensitize cold tumors to existing immunotherapies, effectively turning a biochemical nuance into a potent clinical tool.

## 9 Conclusions and perspectives: Defining the future of ADPRylation and the epigenome

The study of nuclear ADPRylation is undergoing a fundamental paradigm shift. Historically, our understanding was dominated by the rapid, high‐intensity ‘ADPR spray’ of PARylation, a vital but often low‐precision signaling burst typically associated with acute DNA damage. However, as we have detailed here, the field is moving toward a more nuanced appreciation of MARylation as a primary rheostat for nuclear physiology (Fig. 1). This transition marks a shift from broad, scaffold‐based signaling to a model of site‐specific precision, where the modification of a single residue on a histone or co‐regulator can dictate the recruitment of remodeling complexes like SWI/SNF or the enzymatic output of p300 and methyltransferases.

**Fig. 5:**
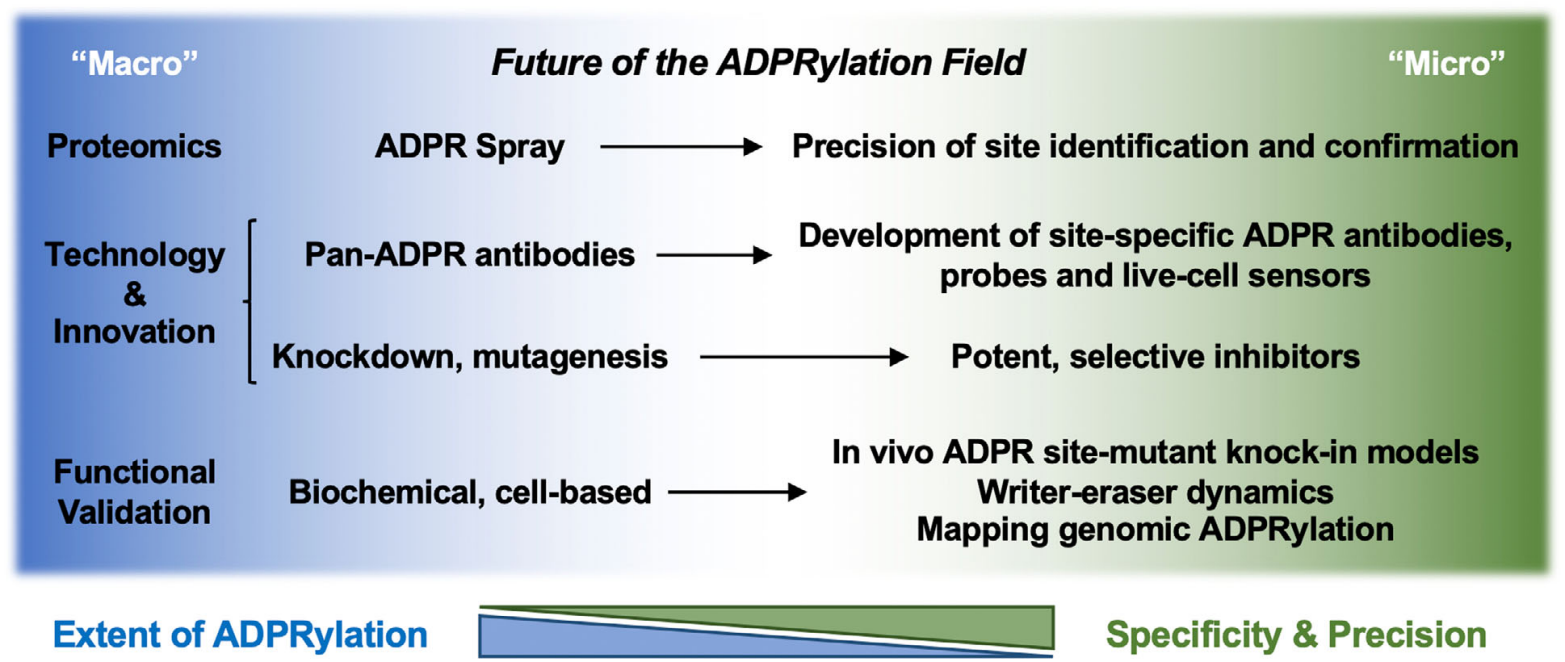
Future of the ADPRylation field. This figure highlights key strategic objectives and future directions required to decode the ADP‐ribose signaling axis. Together, these efforts will enable a transition from a macroscopic view of ADP‐ribose signaling to a high‐resolution understanding of site‐specific ADP‐ribose marks as integral components of the epigenomic landscape. Schematic generated using Microsoft PowerPoint.

To fully decode the impact of ADP‐ribosylation on the epigenome, the field must prioritize the following strategic objectives (Fig. 5):

*Precision identification and confirmation*: We must move beyond global proteomics to identify the specific amino acid sites of ADPRylation on key epigenomic regulators. This requires the continued development of robust mass spectrometry‐based methods to definitively confirm these modification sites under physiological conditions.

*Technological advancement in detection*: The generation of site‐specific ADPR‐peptides and highly sensitive, site‐specific antibodies is paramount. These tools will allow researchers to monitor the accumulation of specific sites of ADPRylation *in vivo* and track the occupancy of specific MAR marks across the genome with the same resolution we currently apply to phosphorylation, acetylation, or methylation. Paired with ADPR site mutants used in genetic models, these antibodies will provide a powerful combination to explore the biology of site‐specific ADPRylation *in vivo*.

*Deciphering the writer‐eraser dynamics*: Determining the precise biochemical mechanisms by which MARTs (writers) and ADP‐ribosyl hydrolases (erasers) target specific sites will be essential to fully understand their biology and their therapeutic potential. Understanding this enzymatic tug‐of‐war is essential for predicting the duration and amplitude of nuclear signaling events.

*Mapping genomic localization*: A complete understanding of how ADPRylation controls the epigenome will require fine mapping of ADPRylation, writers and erasers, and response to chemical inhibitors on a genomic scale. This information will allow functional prediction of specific mechanisms driving gene regulation and genome control.

*Functional validation across scales*: Determining the biological consequences of site‐specific MARylation requires a multi‐scale approach, from rigorous *in vitro* biochemical assays to cell‐based functional studies. Ultimately, the use of ADPR site‐mutant knock‐in models will be the gold standard for defining the *in vivo* necessity of specific modifications in complex biological systems.

*Chemical and synthetic innovation*: The development of small‐molecule inhibitors, synthetic probes, and live‐cell sensors is critical. These tools will enable the real‐time monitoring of ADPRylation dynamics in living tissues, providing a window into how these modifications integrate with cellular metabolism and stress responses.

By transitioning from a ‘macro’ view of ADP‐ribose polymers to a ‘micro’ view of site‐specific mono‐modifications, we will uncover a regulatory layer that is as precise, diverse, and biologically significant as any other hallmark of the epigenome.

## Conflict of interest

WLK is a founder, consultant, and Scientific Advisory Board member for ARase Therapeutics, Inc. WLK is also a coholder of US Patent 9 599 606 covering a set of ADP‐ribose detection reagents, which has been licensed to and is sold by MilliporeSigma.

## Author contributions

CVC and WLK were involved in conceptualization. CVC, CL, and WLK were involved in writing. CVC was involved in graphical elements and figures. CVC and WLK were involved in review and editing.
